# Burnout among after-hours home visit doctors in Australia

**DOI:** 10.1186/s12875-016-0400-8

**Published:** 2016-01-13

**Authors:** Chris O. Ifediora

**Affiliations:** School of Medicine, Griffith University, Gold Coast Campus, Parklands Drive, Southport, QLD 4215 Australia

**Keywords:** Burnout, General practitioners, Family physicians, Doctors, Home visits, After-hours

## Abstract

**Background:**

No previous study had ever looked at Burnout among medical practitioners involved in after-hours house calls (AHHC) in Australia. The growing popularity of AHHC and the high number of overseas-trained doctors involved in it make this a subject of both local and international significance. This study aims to assess the levels of burnout among Australian-based doctors involved in the service.

**Methods:**

This is a quantitative, questionnaire-based survey of all the 300 doctors engaged in AHHC through the National Home Doctor Service (NHDS), Australia’s largest home doctor-visit service providers. The study looked at experiences of the participants over the 12-months period from October 2013 to September 2014. The main outcome measure was the 22-item Maslach Burnout Inventory (MBI). Results were presented as Means and Frequency Percentages.

**Results:**

A total of 168 valid questionnaires out of 300 were returned, giving a 56.0 % response rate. The Total Maslach Mean Scores (MMS) obtained were 15.97 for Emotional Exhaustion (EE), 3.15 for Depersonalization (DP) and 40.39 for Personal Accomplishment (PA), signifying low-level burnouts in all three dimensions of the MBI. This pattern was equally reflected when the Frequency Percentages were analyzed. With this approach, a majority (56.8 %) of the responses were low-level burnout on the EE dimension, while 23.4 and 19.8 % respectively reported medium and high level burnouts. On the DP dimension, 87.6, 6.3 and 6.1 % of the responses were low, moderate and high level burnouts respectively, while the PA dimension recorded 86.4, 9.6 and 4.0 % respectively. Given that on the MBI scale, perceived personal accomplishment has an inverse relationship with burnout, the low-level MMS finding on the PA dimension therefore indicate a commensurate high perception of personal accomplishments.

**Conclusions:**

Burnout levels are low while perceived achievements are high among doctors involved in after-hours house calls in Australia. The survey recommends that future studies be designed to explore the real reasons behind these findings.

## Background

Burnout represents a long term reaction to emotional and interpersonal stress, and is a syndrome that is defined by exhaustion, cynicism and inefficiency [1]. These three components are respectively categorized as Emotional Exhaustion (EE), Depersonalization (DP) and reduced Personal Accomplishment (PA) [1–3]. EE reflects a feeling of “emotional over-extension, exhaustion and depletion” in one’s work, while DP signifies “impersonal, callous, negative and detached” feelings and responses to the recipients of one’s services [4]. PA, on the other hand, describes a feeling of “competence” and “successful achievement” in one’s discipline and work with people [4]. Of the three, EE has been shown to have the most consistent relationship with job stressors [2, 5].

Regardless of demographic factors, all medical specialties are prone to burnout [6], but those caring for patients with chronic and incurable conditions (like General Practitioners and Psychiatrists) are at high risk because they can only offer “care” to patients who need “cure” [3, 7]. For the General practitioners (GPs), a number of other peculiar challenges make them even more prone to burnouts. These peculiarities include their frontline roles with patients, a large turnover rate of their patients, and the lack of buffer offered by the presence of junior medical and ward staff available to their hospital-based colleagues [3, 7]. There is no doubt that these vulnerabilities apply to both the regular-hour, office-based GPs as well as those involved in after-hours house calls (AHHC).

Even though some burnout is necessary for optimum performance [8], the consequences might have some significant practical implications, including absenteeism, productivity problems, job dissatisfaction, lower quality of care, and patient dissatisfaction [9]. In fact, a study [8] reported that, as a consequence of burnout, up to 25 % of surveyed GPs had positively considered leaving the practice, while 45 % wished to retire at 55 years of age. A few other studies discussed next have looked at burnout levels among regular-hour, office-based GPs, but no published study has ever looked at burnout among home-visit, after-hour doctors. A Canadian study published in 2008 [3] found that 42.5 % of family physicians were stressed (with 28.9 % being low-level low-burnout), irrespective of their demographics, while a 1998 British study [10] reported that 52 % of GPs had psychological symptoms, with 60 % admitting to their physical health being affected by their work. In Australia, Murfett and Charman [11] published a study in 2006 that showed that 15 % of GPs report “poor” or “very poor” well-being due to burnout, while another study published in the United States [12] found that, at least, one-in-three doctors experience burnout at any given time. Another British publication showed a higher than average prevalence of burnout among health professionals relative to the general population (28 vs 18 %) [13].

The fact that no previous study has ever looked at burnout levels among doctors involved in AHHC becomes worrisome as one considers the increasing popularity of the industry in Australia. It is on record that the service attended to 1.51 million Australians in 2013 [14], and given its beneficial cost-per-patient ratio over emergency departments [14, 15], the industry is expected to continue to garner government support, and will likely expand in the future. A recent article [15] opined that, apart from this cost-benefit that will likely ensure sustained government support, the popularity and rapid expansion is equally fueled by the fact that patients are happy with the industry as most of the service-providers offer “bulk-billed” services (i.e., accept payments directly from the Australian-government funded universal health insurance system called Medicare), while the doctors involved receive much better remuneration compared to their office-based colleagues. It therefore becomes imperative that burnout among the doctors in the service is analyzed, so as to help practitioners and healthcare managers make better and informed decisions regarding the planning, recruitment and the welfare of doctors. This study aims to achieve this purpose.

A knowledge of Burnout Levels for AHHC would also have international relevance given the high number of overseas-trained doctors (OTDs) in the workforce. A recent survey [16] found that about 71 % of the Australian AHHC workforce obtained their primary medical degrees from overseas, while data from 2011 showed that 56 % of the entire doctors in Australia were OTDs [17]. Even though the reason for the higher number of OTDs in AHHC than the general medical population is not clear, these numbers indicate that medical practice in Australia (including AHHC services) are attractive to OTDs, and the findings from this report might provide some guidance to other OTDs planning to join the workforce. In addition, different countries round the world are at different levels of involvement and development of their AHHC industry (also called Medical Deputizing Services, MDS), and healthcare managers in these countries may wish to look at the findings from this survey as they (re) design their own systems. For instance, it has been speculated that the British government is reviewing the MDS in the United Kingdom (UK) with a view to handing it from Primary Care Trusts (PCTs) directly to GPs [14]. In the same vein, AHHC in Canada is still not developed, with their emergency departments (EDs) and late-opening clinics being over-utilized [14]. Things are different in the Netherlands (and, possibly France), where there is already a National Model of MDS in place in view of the high demands for the service [14]. There is no doubt that, given the diverse AHHC issues in the aforementioned countries, findings from this work would be useful to their healthcare managers and doctors alike in forging ahead.

## Methods

### Setting and participants

The Participants include all primary care doctors (GPs and others) who undertake AHHC through the National Home Doctor Service (NHDS) in Australia. The NHDS is the largest accredited member of the National Association of Medical Deputising Services (NAMDS), the body of all AHHC service providers in Australia [18]. Even though the total number of doctors in the Australian AHHC industry is not known as involvement is free and requires no formal registration apart from being a qualified medical practitioner, it can be safely assumed that a study of NHDS doctors reasonably represents that of the Australian after-hours doctor-community given that over the last few years prior to this study, the NHDS has successfully annexed the largest AHHC clinics in Australian major towns and cities [18].

NHDS have presence in Sydney, the Gold Coast, Adelaide, Brisbane Area (including Sunshine Coast), and Melbourne Area (including Geelong and Canberra) [19]. The terms “Melbourne and Brisbane Areas” reflect the NHDS administrative groupings, and are not based on geographical or political classifications. Each NHDS-location is overseen by a Clinical or General Manager. Private correspondences with the Chief Executive Officer (CEO) of NHDS confirmed that 300 doctors were employed with the company at the time of this survey, making this number the study population. This survey focuses on the 12 months from October 2013 to September 2014.

By definition, after-hours house-call (AHHC) services involves house-visits by doctors outside regular working hours on weekdays (6 pm to 8 am), Saturdays (from 12 noon), Sundays and Public Holidays (all day) [19].

### Questionnaire

The *SurveyMonkey*^*R*^ software was used in the designing and collation of the questionnaire, which was an 11-paged, electronic, anonymized document. The aspect relating to this study covered Pages 1 to 5 of the questionnaire, as well as Pages 9 and 10, with questions presented under different sections (Introduction/Consent, Bio-data, Professional and Burnout Sections). Pages 6 to 8 of the questionnaire were designed to gather information on “Aggression” and “Satisfaction” from the respondents, which were not covered in this work, while Page 11 was the “appreciation” or “thank you” page to the respondents.

The validated 22-item Maslach Burnout Inventory (MBI) was used to assess burnout because of its proven ease of completion, reliability, validity and applicability to GPs [1, 5, 9, 20]. The MBI assesses Burnout by identifying the frequency (how often) with which various feelings occur over a 12-month period. These feelings are grouped into three Dimensions: Emotional Exhaustion, EE (9 questions), Depersonalization, DP (5 questions), and reduced Personal Accomplishment, PA (8 questions), totaling 22 questions. The answers are presented on a 7-point Likert Scale, from “never (0)” to “everyday (6)”. Even though the MBI tool has not been specifically validated for use in the Australian AHHC primary care setting, it has been validated and used for a study involving tens of thousands of Australian doctors and medical students [21], a group that arguably includes those involved in AHHC services. For this reason, therefore, the merit and applicability of the MBI tool to the participants in this study is reasonably justified.

The questionnaire reached the participants as links sent through emails via the respective General Managers in charge of their Locations. This choice of the General Managers was made in other to encourage participation from the respondents and hopefully boost the response rate. However, to avoid possible bias that might arise from this method of distribution, the completed questionnaires were not returned through the General Managers, but were rather sent directly to the researcher through the *SurveyMonkey*^*R*^ tool by clicking the “submit” button on completion of the survey.

A total of two reminders were sent to the respondents at fortnightly intervals after the initial dispatch. Data collection took about 6 weeks, from the end of September 2014 to the middle of November 2014.

### Sample size estimation

The *Sample Size Calculator* tool in the *National Statistical Service* website [22] was used to estimate the minimum number of responses required to give a statistically robust survey. An estimation was done based on a previous study [3] that found 28.9 % prevalence of low-burnout among GPs in Canada. With a population size of 300 in this study, and allowing for an error margin of 5 % with a 95 % Confidence Interval (CI), the minimum required number of responses was found to be 154.

### Analysis

Analysis was with *IBM SPSS Version 22*. For the sake of interpreting burnout, and to ensure that a comprehensive analysis was done, the results were explored, presented and discussed using three different parameters (as guided by previous studies), with 95 % Cis and Standard Errors given were possible.

The first Parameter uses the Percentage Frequencies of each Dimension of Burnout, and compares them to frequencies from previous studies. To achieve this, raw frequencies of response rates were originally obtained for each MBI item, with items on a 7-point Likert Scale (“never” to “everyday”). These were then re-coded into three new groups as described in a previous study [23], and presented as follows

1. Low-level burnout = “Never” *plus* “Few-times-a-year-or-less”;
2. Moderate-level burnout = “Once-a-month” *plus* “Few-times-a-month”;
3. High-level burnout = “Once-a-week” *plus* “Few-times-a-week” *plus* “Every-day”.

The Second Parameter presents the total of the Mean Scores for each of the dimensions (EE, DP and PA), and compares them to the Maslach Reference Ranges. These ranges are classified as Low, Moderate or High-level Burnout. A guide to interpretation as defined by Maslach Reference Ranges [9, 24], is as follows:

1. Emotional Exhaustion: Low = “17 or less”; Moderate = “18-29”; High = “30 or more”.
2. Depersonalization: Low = “5 or Less”; Moderate = “6-11”; High = “12 or More”.
3. Personal Accomplishment: High = “33 or less”; Moderate = “34 to 39”; Low = “40 or More”.

The Third Parameter is related to the second, but uses the Average Mean per Item (not totals) of each Dimension, and compares it with the Maslach Study Sample [25] as a reference.

Results based on these Parameters are all discussed further in the next Section. It should be noted that this paper only looked at the levels of burnout (not associations) among the participants. Due to volume-restrictions, the associations of these burnout parameters with independent variables for doctors involved in AHHC were presented and published separately in another related paper [26].

### Ethical considerations

Ethical clearance was obtained from the Human Research Ethics Committee of the Griffiths University, Australia (GU Ref No: MED/47/14/HREC), prior to commencing the study. Consent was equally mandatory before participants were able to compete the questionnaires.

## Results and discussion

172 respondents returned their questionnaires, but 4 of these did not complete the Basic Bio-Data Section and were excluded from the analysis, leaving 168 valid responses out of the dispatched 300 (56.0 % response rate). This rate is considered modest as most online surveys generally have poor response rates [27], and it surpasses the 154 minimum estimated sample size for the study. Despite the expected low response rates with online questionnaires, this study opted for this method for a number of advantages conferred by the online approach over postal or direct surveys [28]. Firstly, cost was a key consideration given that the survey was self-funded. In addition, the ease of dispatch, collation and follow-up, as well as the convenience for the respondents (important in getting them to respond given the usually busy schedule of doctors) were other key considerations. In any case, the rate from this survey was better than that from a 2012 survey of European GPs, which recorded a 41 % response rate [29], but is poorer than most other similar studies, including that Canada (78 %) [3], the UK (70 to 83 % range) [10, 25, 30, 31], France (64 %) [32], and Switzerland (66 %) [33]. However, all the cited studies depended on postal or directly administered questionnaires, and, as already noted, these generally tend to fare better than the online ones [27].

The basic response characteristics are summarized in Table 1, and include Gender, Age, Specialty, Marital Status, Family Setting, Postgraduate Vocational Status, Hours worked/week, Duration in After-hours, and Country of Primary Degree. At 19.6 %, the proportion of females involved in AHHC in Australia is much less than the 43 % involved in regular-hour general practice [17]. A majority of the respondents were career GPs (84.4 %), with the rest being in other specialties (Physicians, Surgeons, Anesthetists, etc.). Just over half (53.6 %) were in the 40–60 age bracket, with 41.1 % being aged 39 or less and only 5.4 % being over 60 years of age.

**Table 1:** *Summary of the basic statistics of the doctors* involved in after-hours, house calls in Australia

Statistic	Parameters	*N*	%
Gender Valid = 168	Male	135	80.4
	Female	33	19.6
Age Range (Yrs) Valid = 168	39 or less	69	41.1
	40-60	90	53.6
	Over 60	9	5.4
Vocational/Registration status Valid = 137	Vocationally registered (Fellows)	61	44.5
	Non-vocationally registered (Non-fellows)	76	55.5
Primary degree Valid = 160	Australian-trained	45	28.1
	Overseas: New Zealand	6	3.8
	Overseas: other	109	68.1
Specialty Valid = 160	General Practice	135	84.4
	Medical	7	4.4
	Surgical	2	1.3
	Emergency Department	6	3.8
	Others (Paediatricians, Anaesthetists, etc.)	10	6.3
Location of Service Valid = 160	Adelaide	51	31.9
	Brisbane Area (Brisbane and Sunshine Coast)	36	22.6
	Gold Coast	23	14.4
	Melbourne Area (Melbourne, Geelong and Canberra)	31	19.4
	Sydney	17	10.6
	Other (unfixed location)	2	1.3
Duration In After-Hours Valid = 160	<= 2 years	80	50.0
	2–10 years	54	33.8
	>10 years	26	16.3
Hours worked/week Valid = 160	<24 h/week	62	38.8
	24 to 37.5 h/week	47	29.4
	>37.5 h/week	51	31.9
Marital status Valid = 168	Married	140	83.3
	Single	12	2.4
	De facto (Co-habitation & civil partnership)	10	6.0
	Separated	4	2.4
	Widowed	2	1.2
Family setting Valid = 168	Lives with own kids	115	68.5
	Have kids/live with none	17	10.1
	No kids/Live with none	36	21.4

The rest of the results are presented and discussed according the three Burnout parameters identified on the *Analysis Section* above.

### Burnout parameter 1: frequency percentages

i. Emotional Exhaustion (EE)

Table 2 summarizes the frequencies of experiences in the three different dimensions of Burnout. Just under 1-in-5 (19.8 %) and nearly 1-in-4 (23.4 %) of the respondents recorded high-level and moderate-level burnouts respectively on the EE Dimension, leaving well over half (56.8 %) with low-level burnout. Unfortunately, no AHHC study had ever been published to look at Burnout in this regard, but comparisons made to regular-hours, office-based GPs show these AHHC rates are generally more favourable. For instance, a study of 158 office-based, regular-hour Canadian Family Physicians published in 2008 showed 47.9 % of high-level burnout on EE, with a 28.9 % of low burnout [3]. Apart from Canada, findings from a survey of in-hour GPs based in Switzerland [33], the United Kingdom [31], Iran [34] and the entire Europe [29] showed high-level burnouts of 33, 46, 27.3 and 43 % respectively, all higher than the levels in the AHHC doctors reported in this survey. The only Australian study to show relevance was a 1991 survey of regular-hour GPs in in South Australia, which showed that one-third of the 966 surveyed doctors had significant job stress [31]. However, the particular dimension for that survey were not reported.

**Table 2:** Frequencies, Means and Averages of experiences in the various dimensions of Stress among after-hours, house call doctors in Australia

Burnout range/definition (Number of experiences)	Total experiences	Percentage frequency of experiences	
1. Frequencies of Emotional Exhaustion (EE)			
Low = Never (409) + Few-times-per-year (301)	710	56.8 %	
Medium = Once-per-Month-or-less (126) + Few-times-per-month (167)	293	23.4 %	
High = Once-per-Week (101) + Few-times-per-week (88) + Everyday (58)	247	19.8 %	
Totals for EE (TEE)	1,250	100.0 %	
2. Frequencies of Depersonalization (DP)			
Low = Never (504) + Few-times-per-year (109)	613	87.6 %	
Medium = Once-per-month-or-less (29) + Few-times-per-month (15)	44	6.3 %	
High = Once-per-week (10) + Few-times-per-week (25) + Everyday (8)	43	6.1 %	
Totals for DP (TDP)	700	100.0 %	
3. Frequencies of Personal Accomplishment (PA)			
High (Low PA) = Never (7) + Few-times-per-year (38)	45	4.0 %	
Medium = Once-per-month-or-less (22) + Few-times-per-month (85)	107	9.6 %	
Low (High PA) = Once-per-week (72) + Few-times-per-week (304) + Everyday (588)	964	86.4 %	
Totals for PA (TPA)	1116	100.0 %	
4. Averages and Means			
Dimension	Total Mean Scores	Average Scores (out of 6)	Interpretation
^a^Emotional exhaustion, EE (9 items)	15.97	1.77	Low-level Stress
^b^Depersonalization, DP (5 items)	3.15	0.63	Low-level Stress
^c^Personal accomplishment (8 items)	40.39	5.05	Low-level Stress

NB: ^a^Emotional Exhaustion: 17 or less = Low Level; 18 to 29 = Moderate Level; 30 or more = High Level ^b^Depersonalization: 5 or Less = Low Level; 6 to 11 = Moderate Level; 12 or More = High Level ^c^Personal Accomplishment: 33 or less = High Level; 34 to 39 Moderate Level; 40 or More = Low Level)

Still on percentage rates, the respective rates for the nine individual items of EE are summarized on Table 3. These items include experiences of the following when dealing with patients: “feeling emotionally drained”, “feeling used up”, “feeling fatigued in the mornings”, “driving round is a strain”, “feeling burned out”, “feeling frustrated”, “working too hard”, “stressed working with people”, and “feeling at the end of the rope”. From the Table 3, it is obvious majority of the respondents (between 4-in-10 to just over 8-in-10 practitioners) reported either “a few” or “no” feelings of any of the EE items over the entire 12-month surveyed, while a minority (between 1-in-10 to 3-in-10) expressed a “daily” to “once-a-week” feelings of these same experiences.

**Table 3:** Response rates to “Emotional Exhaustion” items among after-hours, house call doctors in Australia

	Emotional exhaustion items	Low-level burnout never + Few times Per Year (%)	Moderate burnout once per month Or less + Few times per Month (%)	High-level burnout once per week + Few times per week + Everyday (%)	Mean: out of 6 (95 % CI)	Standard error of mean
1	Feeling emotionally drained (Valid 140)	60 (42.8)	49 (35.0)	31 (22.1)	2.20 (1.92–2.48)	0.14
2	Feeling Used Up (Valid 140)	55 (39.2)	43 (30.7)	42 (30.0)	2.47 (2.16–2.78	0.16
3	Feeling fatigued in the mornings (Valid 140)	65 (46.4)	44 (31.4)	31 (22.1)	2.24 (1.93–2.55)	0.16
4	Driving round is a strain (Valid 139)	68 (49.0)	39 (28.1)	32 (23.0)	2.02 (1.70–2.35)	0.16
5	Feeling burned out (Valid 139)	83 (59.7)	26 (18.8)	30 (21.6)	1.76 (1.45–2.08)	0.16
6	Feeling frustrated (Valid 139))	85 (61.2)	30 (21.2)	24 (17.3)	1.61 (1.31–1.91)	0.15
7	Working too hard (Valid 137)	83 (60.6)	26 (19.0)	28 (20.4)	1.73 (1.42–2.04)	0.16
8	Stressed working with people (Valid 138)	98 (71.0)	23 (16.6)	17 (12.3)	1.22 (1.93–2.55)	0.14
9	Feeling at the end of the rope (Valid 140)	113 (81.9)	13 (9.4)	12 (8.7)	0.72 (0.46–0.97)	0.13
	TOTALS (%)	710 (56.8)	293 (23.4)	247 (19.8)	15.97	

Explanations for these low-level and infrequent feelings of emotional exhaustion among the respondents may be related to the way the AHHC services is organized by the NHDS. Private correspondences with the management of the company revealed that, even though most weekday (and night) schedules commence at 6 pm, the involved doctors work in shifts, with majority starting by 6 pm and finishing by 1 am (or earlier in most cases) of the next morning. A fresh set of doctors, who did not start with the others at 6 pm, take over from 1 am, and then work till 8 am of the same morning. The Weekends and public holidays are slightly different, with the practitioners working 10-h shifts, which are split into two blocks of 5-h, with a 2-h break in between. This time schedule will no doubt allow most practitioners to have good rests overnight or in the daytime as the case might be, thereby limiting burnout with regards to most items of the EE dimension. Burnout may further be limited by the fact the NHDS allow most of their doctors to negotiate schedules that are convenient for them. The doctors are also allowed to get involved full-time or part-time. These flexibilities will allow the doctors to work take up shifts that suit them and these will no doubt minimize burnout in the industry. A final possible explanation may be linked to the fact that the doctors are allowed to use chauffeurs or chaperones if so desired, and most of the driving to see patients are at less busy times of the day or nights, when the roads are not very congested. These may limit the burnouts related to driving around or feeling fatigued. In fact, such a measure has indeed been found to be associated with reduced burnout [26] and increased satisfaction [35] when working in AHHC, findings which substantiate these theories. It is important to note that these explanations are theories, and a well-designed qualitative study may be useful in exploring these, as well as other possibilities.

ii. Depersonalization (DP)

On the DP Dimension, only 6.1 % of the respondents reported have high-level burnout, while 6.3 % indicated moderate levels. 87.6 % of burnouts on this scale were low-level (Table 2). These rates compared favorably with a Canadian study [3], which recorded 46.3 % high burnout and 30.6 % low burnout on DP respectively. The rates also appear better when compared to results from studies in Switzerland [33], the UK [31], Iran [34] and Europe [29], with reports of 28, 42, 94.7 and 35 % high-level burnouts respectively. These studies are all in regular-hour GP practices, as no previous study had ever been published on AHHC in this regard, and so caution should be applied when interpreting these comparisons.

Table 4 summarizes the frequencies of the experiences of the five individual items on the DP dimension. As a reminder, these items include feelings of “treating patients impersonally” “being callous to patients” “not caring what happened to patients” “becoming emotionally hardened” and “feeling blamed by the patients”. The table that 8-in-10 to 9-in-10 respondents have either never experienced these feelings at all, or have only had a few experiences of them over the 12-month period under survey. On the other hand, less than 1-in-10 practitioner admits to having these feelings on a daily or at least once-weekly basis in the same period.

**Table 4:** Response rates to “Depersonalization” items among after-hours, house call doctors in Australia

	Depersonalization items	Never + Few times Per Year (%)	Once per month Or less + Few times per Month (%)	Once Per Week + Few Times per Week + Everyday (%)	Mean: out of 6 (95 %CI)	Standard Error (SE) of Mean
1	Treats patients Impersonally (Valid 140)	114 (85.0)	11 (7.9)	10 (7.2)	0.66 (0.42–.91)	0.12
2	Became Callous to Patients (Valid 140)	122 (86.9)	12 (8.6)	6 (4.3)	0.58 (0.35–.80)	0.11
3	Became Emotionally Hardened (Valid 140)	123 (87.9)	9 (6.4)	8 (5.7)	0.68 (0.45–.90)	0.11
4	Does not care what happens to Patients (Valid 140)	129 (92.1)	5 (3.6)	6 (4.3)	0.41 (0.21–.68)	0.10
5	Feels blamed by Patients (Valid 140)	120 (85.9)	7 (5.0)	13 (9.3)	0.82 (0.56–.07)	0.13
	TOTALS (%)	613 (87.6)	44 (6.3)	43 (6.1)	3.15	

The possible reasons for these low-level and infrequent feelings of depersonalizations may be related to the lack of the need for continuity of patient-care required of doctors involved in AHHC. This is so because, most follow-ups after AHHC consultations are with the patients’ regular GPs, meaning that the major responsibilities of care in the service are passed on to these GPs, which, in most cases are office-based, regular-hour practitioners. With this burden off the AHHC doctors, it will not come as a surprise that the level of burnout associated with the DP items mentioned above are less on these doctors, as those with ongoing care of their patients would be expected to be more vulnerable to these feelings.

iii. Personal Accomplishment (PA)

In accordance with the DP dimension, the respondents reported a very high percentage of low-level burnout (equivalent to high-level achievement) on the PA dimension (86.4 %), leaving 4.0 and 9.6 % respectively on the high and moderate levels (Table 2). Compared to previous studies of regular-hour doctors, the closest low-level burnout on the PA dimension was a study of GPs in Iran, with a 73.7 % rate [34], but low-level burnout in other countries appear much lower. Examples include results from Switzerland (20 %) [33], the UK (34 %) [31] and the entire Europe (32 %) [29]. These comparisons show that the AHHC in Australia have a lower level of burnout but higher perceived achievements relative to the regular-hour GP jobs in these countries.

An analysis of the frequencies of the experiences of the eight individual items in the PA dimension, summarized in Table 5, indicate very high percentages for each. The concerned items are the feelings of “being effective with the patients’ problems”, “having positive influences on the patients”, “easily creating relaxed atmospheres with them”, and “being calm with their emotional problems”. Other items include the feeling of “easily of understanding the patients”, “feeling energetic”, “feeling exhilarated when dealing with patients”, and a feeling of an “accomplishment of worthwhile things while on the job”. As shown in in Table 5, between 7-in-10 and 9-in-10 practitioners experienced these feelings daily or at least once-per-week, while a handful (less than 1-in-10) of them either did not report experiencing these feelings at all, or reported them only a few times a year.

**Table 5:** Response rates to Perceived Personal Accomplishment items among after-hours, house call doctors in Australia

	Personal accomplishment items	Never + Few times per year (%)	Once per month Or less + Few times per month (%)	Once Per Week + Few times per week + Everyday (%)	Mean: out of 6 (95 % CI)	Standard error of mean
1	Easily Understands Patients (Valid 140)	5 (3.4)	13 (9.3)	122 (87.2)	5.25 (5.03–5.48)	0.11
2	Effective with Patient’s problems (Valid 138)	1 (0.7)	4 (2.9)	133 (96.4)	5.58 (5.45–5.72)	0.07
3	Positively Influence patients (Valid 139)	4 (2.9)	10 (7.2)	125 (89.9)	5.15 (4.94–5.35)	0.10
4	Feels Energetic (Valid 140)	7 (5.0)	25 (17.8)	108 (77.1)	4.61 (4.35–4.86)	0.13
5	Easily creates relaxed atmosphere (Valid 140)	1 (0.7)	11 (7.8)	128 (91.4)	5.42 (5.23–5.60)	0.09
6	Feels Exhilarated (Valid 139)	11 (7.9)	19 (13.7)	109 (78.4)	4.52 (4.24–4.80)	0.14
7	Accomplished worthwhile things (Valid 140)	12 (8.6)	20 (14.3)	108 (77.2)	4.58 (4.30–4.87)	0.14
8	Calm with emotional problems (Valid 140)	4 (2.9)	5 (3.6)	131 (93.6)	5.28 (5.09–5.48)	0.10
	TOTALS (%)	45 (4.0)	107 (9.6)	964 (86.4)	4039	

The real reasons behind the findings on the PA dimension is not very clear. Most of the items reflect how the doctors feel they relate to the patients. The high levels recorded in these might be related to the independence enjoyed by the doctors involved in this service, given that they mostly work alone. Such independence may allow them display a more personal and relaxed consultation when seeing these patients. In addition, private discussions with the NHDS indicate that the doctors involved in AHHC do not see as many patients per hour compared to those in regular-hour office jobs, meaning that they are likely to spend more per patient. This may result in a higher level of time spent in understanding, calming, and influencing their patients. This explanation is merely a theory, and a new survey may be the best way to explore these findings. However, the high level regarding a feeling by doctors that they had “accomplished more worthwhile things” while in AHHC may be related to the fact that they get paid at a higher rate per consultation compared to their office-based colleagues [15], meaning that they might have more disposable income than their other colleagues.

### Burnout parameters 2: total means

i. Emotional Exhaustion (EE) and Depersonalization (DP)

A Maslach Mean Total Scores of 15.97 was recorded on the EE Dimension, while On Depersonalization (DP), it was 3.15 (Table 2). These both indicate that burnout for the EE and DP dimensions were in the low-level ranges, concurring with findings from Parameter 1. Again, these scores appear to be better than scores from office-based studies of GPs in Southern Californian (which reported with means of 24.4 for EE and 9.4 for DP) [9] as well as those from Switzerland (which recorded means of 22.3 for EE and 7.4 to DP) [33]. As can be seen, these two cited studies are both in the moderate burnout range, but caution is advised while comparing these results given the differences in the working conditions and the countries involved. Even though the exact reasons for these findings are not obvious, the same explanations given for the findings on the EE and DP dimensions using the “burnout parameter 1” section may still apply, but further studies may be required for full exploration.

ii. Personal Accomplishment (PA)

A score of 40.39 was recorded for this dimension, consistent with low level of burn-out and a high-level feeling of personal accomplishment (Table 2). This is similar to the level from the already-cited a survey of office-based family practitioners in Southern California (40.1) [9], and GPs in Switzerland (39.4) [33]. These indicate that the perceived achievements of GPs involved in AHHC in Australia are not only high, but are comparable to those elsewhere.

### Burnout parameters 3: average means

An analysis of the Average Mean Score for each Dimension, the third Parameter used by this study to assess Burnout, also revealed similar findings as the second and first. For reference, the Maslach Study Sample recorded Average Means (out of 6) of 2.47 EE; 1.42 for DP and 4.57 for PA [25]. However, findings from this study regarding AHHC doctors (Table 2), found Means of 1.77 for EE and 0.63 for DP, indicating lower averages of burnout on these dimensions relative to the Maslach Reference Means. It equally recorded a higher value for PA (5.05) relative to the Maslach Reference, indicating a higher level of perceived personal accomplishment (low burnout) among the AHHC doctors. Only one other study was found in the literature reporting its findings with this same Parameter. A 1995 study of GPs in Northampton, UK [25], found Means of 2.9, 1.95 and 4.36 respectively for EE, DP and PA which indicate higher level burnout in the surveyed British doctors. Explanations for these interesting findings are in line with those already proffered under the “burnout parameter 1”, but as already indicated, future studies may be needed for full exploration.

Overall, the foregoing discussion have revealed that burnout levels are relatively low among primary care practitioners involved in after-hours house calls in Australia. The reasons behind these are not clear, given that this study was not designed to answer this question, but the voluntary nature of the job in Australia might be a factor, given that those engaged in the service might be those that really enjoy doing it. In addition, the flexibility of the NHDS to their staff, allowing them to choose where to work, what hours to do, and so on, might equally be a factor. However, given the significant nature of the findings of this work, it is hoped that future studies will take up the challenge of unravelling the real reasons behind these relatively low-level burnouts. It should be noted that, even though it was not reported in this paper, a related article regarding the same study population [26] have established that reduced burnout in AHHC is significantly associated with the adoption of self-protection measures while on the job, as well as the attainment of postgraduate fellowships (vocational registration), working less than 24 h per week, being in legally recognised partnerships, and being male, while increased burnout in AHHC was linked to being under 40 years of age, obtaining primary medical degrees from Australia (as opposed to overseas), and practitioners who have general practice as a career (as against being in other specialties).

### Study strength(s) and weakness (es)

The use of a standard, globally acceptable tool to assess Burnout in this survey is a clear strength, as the validity and reliability of the survey tool is not in doubt. Unfortunately, it is acknowledged that a higher response rate would have boosted the generalizability of the findings. Nevertheless, given that 168 responses received for this study exceeded the 154 estimated to give a statistically robust study (please see the section on Sample Size Estimation), this slight limitation may have limited impact after all.

## Conclusions

This study concludes that the levels of burnout among primary care doctors involved in after-hour house calls in Australia are mainly low-level across all dimensions of Emotional Exhaustion, Depersonalization, and Perceived Personal Accomplishment. The level of Perceived Personal Accomplishment among doctors in AHHC is either at the same level, or better than, levels among GPs elsewhere.

## Recommendations

This study recommends that future research explore the real reasons why Australian primary-care physicians involved in AHHC have less burn-out levels than their counterparts in regular-hours, office-based jobs. This knowledge may be useful to in the planning, staff-management and appraisals required for the administration of the after-hour services, particularly for overseas countries interested in the service. Adequate policies should also remain in place to improve on this current trend among AHHC services in Australia.

## Abbreviations

AHHC: After-hours house call:
CEO: Chief Executive Officer:
CI: Confidence interval:
DP: Depersonalization:
ED: Emergency department:
EE: Emergency Department:
GP: General Practitioner:
GU: Gold Coast University:
HREC: Human Research Ethics Committee:
IBM: International Business Machines:
NHDS: National Home Doctor Service:
MBI: Maslach Burnout Inventory:
MDS: Medical deputizing service:
OTD: Overseas-trained doctor:
PA: Personal Accomplishment:
SE: Standard Error:
SPSS: Statistical Package for the Social Sciences:
UK: United Kingdom
